# Soluble triggering receptor on myeloid cells-1 is expressed in the course of non-infectious inflammation after traumatic lung contusion: a prospective cohort study

**DOI:** 10.1186/cc10141

**Published:** 2011-04-15

**Authors:** Tobias M Bingold, Barbara Pullmann, Sven Sartorius, Emanuel V Geiger, Ingo Marzi, Kai Zacharowski, Heimo Wissing, Bertram Scheller

**Affiliations:** 1 Clinic of Anaesthesiology, Intensive Care Medicine and Pain Therapy, University Hospital Frankfurt am Main, Theodor Stern Kai 7, 60590 Frankfurt am Main, Germany; 2 Department of Trauma, Hand and Reconstructive Surgery, University Hospital of Frankfurt, Theodor Stern Kai 7, 60590 Frankfurt am Main, Germany; 3 Clinic of Anaesthesia, Intensive Care Medicine and Pain Therapy, Clinic of Barmherzige Brüder Montabaur, Koblenzer Straße 1, 56410 Montabaur, Germany

## Abstract

**Introduction:**

The triggering receptor expressed on myeloid cells-1 (TREM-1) is known to be expressed during bacterial infections. We investigated whether TREM-1 is also expressed in non-infectious inflammation following traumatic lung contusion.

**Methods:**

In a study population of 45 adult patients with multiple trauma and lung contusion, we obtained bronchoalveolar lavage (BAL) (blind suctioning of 20 ml NaCl (0.9%) via jet catheter) and collected blood samples at two time points (16 hours and 40 hours) after trauma. *Post hoc *patients were assigned to one of four groups radiologically classified according to the severity of lung contusion based on the initial chest tomography. Concentration of soluble TREM-1 (sTREM-1) and bacterial growth were determined in the BAL. sTREM-1, IL-6, IL-10, lipopolysaccharide binding protein, procalcitonin, C-reactive protein and leukocyte count were assessed in blood samples. Pulmonary function was evaluated by the paO_2_/FiO_2 _ratio.

**Results:**

Three patients were excluded due to positive bacterial growth in the initial BAL. In 42 patients the severity of lung contusion correlated with the levels of sTREM-1 16 hours and 40 hours after trauma. sTREM-1 levels were significantly (*P *< 0.01) elevated in patients with severe contusion (2,184 pg/ml (620 to 4,000 pg/ml)) in comparison with patients with mild (339 pg/ml (135 to 731 pg/ml)) or no (217 pg/ml (97 to 701 pg/ml)) contusion 40 hours following trauma. At both time points the paO_2_/FiO_2 _ratio correlated negatively with sTREM-1 levels (Spearman correlation coefficient = -0.446, *P *< 0.01).

**Conclusions:**

sTREM-1 levels are elevated in the BAL of patients following pulmonary contusion. Furthermore, the levels of sTREM-1 in the BAL correlate well with both the severity of radiological pulmonary tissue damage and functional impairment of gas exchange (paO_2_/FiO_2 _ratio).

## Introduction

Triggering receptor expressed on myeloid cells-1 (TREM-1) belongs to the immunoglobulin superfamily and is expressed on the surface of myeloid cells (for example, neutrophils). The receptor mediates the inflammatory response to infectious microorganisms by pathogen-associated molecular patterns [1] and might be activated in Toll-like receptor (TLR)-dependent or TLR-independent fashion [2]. A recent meta-analysis of 73 studies confirmed that the soluble form of this receptor (sTREM-1) is a reliable biomarker for bacterial infections [3].

The study by Porfyridis and colleagues showed that the expression of TREM-1 on neutrophils and on monocytes and of sTREM-1 in serum are reliable diagnostic markers of community-acquired pneumonia [4], whilst other studies showed that sTREM-1 in serum is also increased in ventilator-associated pneumonia and sepsis [5,6].

The presence of sTREM-1 in bronchoalveolar fluid of mechanically ventilated patients is a strong predictor for the presence of pneumonia with a cut-off value of 230 pg/ml to predict acute inflammatory changes in response to bacterial infestation [7,8].

Abacterial inflammation is observed in almost 30% of patients with multiple trauma associated with pulmonary contusion [9]. Pulmonary contusion results in disruption of the epithelial and endothelial cell lining of the lung, impairment of the alveolar-capillary barrier, an activation of the innate inflammatory response and subsequent recruitment of leukocytes, monocytes and tissue macrophages [9]. The resulting self-propagating inflammation within the alveolar space might cause devastating lung injury and is associated with a significant mortality.

Given the role of sTREM-1 as a biological marker for an inflammatory response within the lung, we pursued the hypothesis that the inflammation induced by severe trauma might lead to the expression of sTREM-1 within pulmonary tissue. In addition, we correlated the levels of sTREM-1 to the clinical course of pulmonary function and the severity of lung contusion as assessed by a scoring system based on initial CT scans.

## Materials and methods

We included patients admitted to our ICU between 2007 and 2009 suffering from multiple trauma. According to a standardised emergency room protocol, a multislice CT scan was performed in all trauma patients on admission to the hospital. Patients were screened for study enrolment on admission to the ICU. We included patients >18 years of age with multiple trauma and an indication for kinetic therapy (Rotorest®; KCI GmbH, Wiesbaden, Germany), with visible lung contusion in the initial CT scan and/or Abbreviated Injury Scale thorax >3. Informed consent was obtained from their next of kin. Patients were excluded if no informed consent could be obtained or patient bodyweight exceeded 120 kg. Three patients with a positive result for intracellular organisms or a quantitative culture ≥10^4 ^colony-forming units per millilitre in any bronchoalveolar lavage (BAL) collected were therefore excluded from the study population.

Clinical data were documented according to the German Trauma Registry Data Set, which allows quality control and outcome parameters [10]. In addition, every patient was scored for severity of trauma using the Abbreviated Injury Scale (2008 update; Association for Advancement of Automatic Medicine, Barrington, IL, USA) and the Injury Severity Score [11].

In total, 42 patients participated in this prospective cohort study. The study was approved by the local ethics committee (Ethik-Kommission, Johann Wolfgang Goethe-Universität Frankfurt am Main).

### Clinical treatment and data sampling

Following initial trauma care and surgical intervention, patients were mechanically ventilated (Evita4/XL; Draeger, Lübeck, Germany) and subjected to kinetic therapy using a Rotorest® bed (KCI GmbH) for a minimum of 48 hours. Weaning protocols were designed to keep a positive end-expiratory pressure level of 15 mbar with the aim of weaning the patients to spontaneous ventilation within the initial 24 hours. Weaning to spontaneous ventilation mode was realised by successively minimising ventilator support from assisted spontaneous breathing to pressure support of 0 to 15 mbar or to proportional pressure support of 0 to 4 ml/mbar//mbar/l*sec.

All patients received antibiotic treatment at admission to the emergency room (cefuroxime 1.5 g). In the ICU 39 out of 42 patients received further anti-infective treatment due to open fractures, severe skin lesions (penicillin or cefuroxime) or open brain trauma (meropeneme).

BAL samples were collected 16 hours and 40 hours following trauma. An AeroJet catheter (Covidien GmbH, Neustadt, Bayern, Deutschland), containing two lumina, was inserted via the endotracheal tube under sterile conditions. Then 20 ml NaCl (0.9%) were administered into the lung via the flushing line followed by suction to obtain a fluid sample. The BAL samples were divided into two parts: one portion was analysed microscopically and cultured for microbiological analysis; the second portion was centrifuged (Eppendorf Centrifuge 5702R; Eppendorf AG, Hamburg, Germany) for 15 minutes at 4°C and the resulting supernatant stored at -80°C. Positive BAL was defined either as the presence of cells containing intracellular organisms or a quantitative culture ≥10^4 ^colony-forming units per millilitre of BAL. Levels of sTREM-1 in BAL supernatant were determined by ELISA (Human TREM-1 Quantikine ELISA Kit, version DTRM10B; R&D Systems, Minneapolis, MN, USA).

Vital signs as well as the potential occurrence of pneumonia, systemic inflammatory response syndrome or sepsis were documented daily. Routine blood samples were taken once a day via the arterial line and were centrifuged immediately, with the resulting serum aliquots stored at -80°C. IL-6, IL-10 and lipopolysaccharide binding protein (LBP) were determined in the serum using Immulite 2000 (Siemens Medical Solutions Diagnostics GmbH, Bad Nauheim, Germany).

sTREM-1 in serum was analysed in the same way as the BAL, with ELISA (Human TREM-1 Quantikine ELISA Kit, version DTRM10B; R&D Systems).

Patients were classified *post hoc *according to the severity of lung contusion based on the initial CT scan by two independent physicians. Classification was set by visual quantification of the amount of lung contusion (area of high density of lung tissue), ventral and dorsal damage, and one or both sides affected. Four classes of severity of lung contusion resulted: no lung contusion - no signs of lung tissue damage in the initial CT scan; mild lung contusion - moderate ventral or dorsal damage of lung tissue on one side of the lung; moderate lung contusion - moderate ventral and dorsal damage of lung tissue, or dorsal or ventral severe damage on one side of the lung; and severe lung contusion - multiple contusions on one or both sides of the lung.

### Statistical analysis

Statistical analysis was performed using Sigma Plot 11.0 (Systat Software, Inc., San Jose, CA, USA). All data were tested for normal distribution (Shapiro-Wilk test). Data with a negative test for normal distribution are presented as the median with the 25 to 75% range. Normally distributed values are presented as the mean ± standard deviation. A two-tailed *P *value < 0.05 was considered statistically significant.

A correlation between sTREM-1 and the paO_2_/FiO_2 _ratio or lung contusion score was assessed using Spearman's correlation test. Analysis of variance on ranks was performed for sTREM-1 in dependency on the severity of lung contusion and length of stay (LOS) in the ICU (Kruskal-Wallis test; pairwise multiple comparisons were corrected with Dunn's method). Paired multiple comparisons (IL-6/LBP) were corrected using the Bonferroni test (α = 0.05).

## Results

### Patients

We included 45 patients with multiple trauma in the study population. BAL samples were collected in 45 patients at two time points (16 hours and 40 hours following trauma). In three patients the BALs demonstrated >10^4 ^colony-forming units per millilitre, and these patients were excluded from the study cohort. The demographic data for the remaining 42 patients are shown in Table 1.

**Table 1:** Demographics of the study population, scoring for severity of illness and clinical outcome

	All	Lung contusion at initial CT scan				
		None	Mild	Moderate	Severe	*P *value
*N*	42	8	8	9	17	
Median age (years)	36	31.5	44.2	37.0	34.5	NS
Gender (*n*)						
Male	33	6	6	8	13	
Female	9	2	2	1	4	
Body mass index	24.7	24.6	23.8	23.1	24.9	NS
Injury Severity Score (median)	31	27	34	26	36	NS
APACHE II score (median)	10.2	10	8.5	7	8	NS
SAPS II (median)	23.7	16.5	24.5	17	24	NS
Length of stay (days)						
ICU	12.7	10	10	8	14	>0.001
Hospital	27.7	24.5	27.5	22	27	NS
Rotorest^®a^	5.0	5.5	5.0	4.0	6	NS
Mortality (*n*)						
ICU	1	1	0	0	0	NS
Hospital	1	1	0	0	0	NS
paO_2_/FiO_2_		480	500	463	403	<0.05

APACHE, Acute Physiology and Chronic Health Evaluation; NS, not significant; SAPS, Simplified Acute Physiology Score. ^a^Length of kinetic treatment with Rotorest® bed.

sTREM-1 levels in BAL samples were compared with the radiological classified severity of lung contusion at two time points post traumatic injury (16 hours and 40 hours post trauma). Increasing severity of trauma correlated with mean values of sTREM-1 levels (*P *< 0.01, Spearman Rank order correlation (Rs) = 0.461 for 16 hours following trauma; and *P *< 0.001, Rs = 0.582 for 40 hours following trauma, respectively).

**Figure 1:**
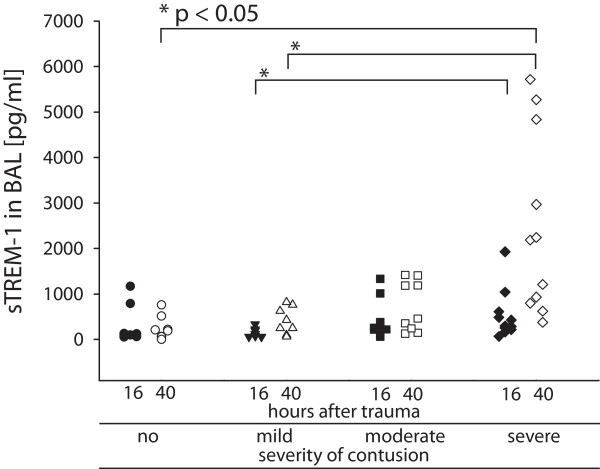
**Soluble triggering receptor correlated with severity of lung contusion after trauma**. Soluble triggering receptor on myeloid cells-1 (sTREM-1) levels in bronchoalveolar lavage (BAL) 16 hours after trauma (solid symbols) and 40 hours after trauma (white symbols) after lung contusion, grouped by severity of lung contusion (based on initial CT scan) in a semi-logarithmic scale. sTREM-1 levels correlated at both time points with increasing severity of contusion (16 hours: *P *< 0.01, correlation coefficient = 0.461 (Spearman rank order correlation); and 40 hours: *P *< 0.001, correlation coefficient = 0.582). The differences in sTREM-1 levels in BAL 16 hours and 40 hours after trauma were significant between the groups with no contusion (only 40 hours after trauma) and with mild versus severe lung contusion (Dunn's method). Differences in sTREM-1 levels additionally reached significance between day 1 and day 2 for the group with severe contusion (*P *< 0.05, Wilcoxon signed rank test).

Sixteen hours after trauma, the median values (range) of sTREM-1 levels in BAL samples were 113 pg/ml (56 to 1,169 pg/ml) in the group with no contusion, 119 pg/ml (62 to 383 pg/ml) in the group with mild contusion, 258 pg/ml (62 to 1,332 pg/ml) in the group with moderate contusion and 299 pg/ml (68 to 1,929 pg/ml) in the group with severe contusion. Forty hours after trauma, the median values (range) of sTREM-1 levels in BAL samples were 217 pg/ml (62 to 1,384 pg/ml) in the group with no contusion, 339 pg/ml (82 to 819 pg/ml) in the group with mild contusion, 459 pg/ml (129 to 1,417 pg/ml) in the group with moderate contusion and 2,184 pg/ml (142 to 5,715 pg/ml) in the group with severe contusion. sTREM-1 values between groups of severity of lung contusion were tested to be significantly different (*P *< 0.05, Kruskal-Wallis test) 16 hours and 40 hours following trauma (Figure 1).

The paO_2_/FiO_2 _ratio at bedside is established to estimate the severity of oxygenation impairment following lung injury. We therefore correlated paO_2_/FiO_2 _with sTREM-1 in BAL. Patients were ventilated at this time point with positive end-expiratory pressure of 15 mbar. There was a negative correlation between sTREM-1 values and the paO_2_/FiO_2 _ratio (Rs = -0.446, *P *< 0.01 for 16 hours following trauma; and Rs = -0.425, *P *< 0.01 for 40 hours following trauma). sTREM-1 values in BAL were not correlated with disease severity (Injury Severity Score, Abbreviated Injury Scale, Acute Physiology and Chronic Health Evaluation II, Simplified Acute Physiology Score II) and LOS of patients in the ICU.

The LOS of patients in the ICU was significantly longer in patients with severe lung contusion (*P *< 0.05, Kruskal-Wallis test) (Table 1). Severity of lung contusion had no significant influence on LOS in the hospital. There was no correlation between LOS in the ICU and sTREM-1 values in BAL or the paO_2_/FiO_2 _ratio.

Additionally we measured sTREM-1 in serum. sTREM-1 values in serum are elevated both 16 hours and 40 hours following trauma, but showed no significant differences between groups of severity of lung contusion or severity of disease. Furthermore there was no correlation to LOS in the ICU or LOS in the hospital.

Data for patients with systemic inflammatory response syndrome and for patients without systemic inflammatory response syndrome also showed no significant difference during the first 40 hours. Within the first 40 hours none of the patients was diagnosed with sepsis or suspected to suffer from an infection of any kind.

### Cytokines

Cytokine levels in blood serum (IL-6, IL-10, LBP, C-reactive protein, procalcitonin, leukocyte count) showed a typical kinetic profile after trauma and were elevated both at 16 hours and 40 hours after trauma.

Cytokine levels showed no correlation to sTREM-1 values in BAL or serum 16 hours following trauma. Forty hours following trauma, IL-10 correlated negatively and the leukocyte count as well as C-reactive protein and LBP correlated positively with the values of sTREM-1 (IL-10: Rs = -0.376, *P *< 0.05; leukocyte count: Rs = 0.313, *P *< 0.05; C-reactive protein: Rs = 0.410, *P *< 0.01; LBP: Rs = 0.403, *P *< 0.05 (Spearman rank order correlation)) (Table 2).

**Table 2:** Cytokine and sTREM-1 values in serum 16 hours and 40 hours following trauma

	All		Lung contusion at initial CT scan							
	16 hours	40 hours	None		Mild		Moderate		Severe	
			16 hours	40 hours	16 hours	40 hours	16 hours	40 hours	16 hours	40 hours
IL-6	182 (112 to 363)	134* (70 to 282)	276 (185 to 443)	348 (120 to 600)	137 (66 to 223)	90 (54 to 163)	184 (108 to 225)	104 (66 to 209)	183 (132 to 418)	157* (64 to 290)
IL-10	14 (6 to 40)	5* (5 to 11)	26 (12 to 41)	16 (7 to 47)	21 (13 to 49)	5* (4 to 11)	10 (6 to 42)	5 (5 to 11)	12 (5 to 36)	5* (4 to 6)
PCT	0.47 (0.11 to 1.11)	0.45 (0.14 to 2.15)	0.1 (0.09 to 0.55)	0.3 (0.15 to 1.23)	0.15 (0.08 to 1.06)	0.16 (0.11 to 1.54)	0.56 (0.17 to 0.68)	0.41 (0.10 to 0.63)	0.90 (0.36 to 6.01)	1.1 0.27 to 5.10)
CRP	2.4 (1.2 to 4.6)	10.4* (7.3 to 14.2)	1.2 (0.8 to 2.8)	8.0 (6.3 to 11.5)	2.0 (1.5 to 5.7)	8.4* (7.2 to 14.3)	2.4 (1.5 to 3.7)	10.9* (8.8 to 13.6)	3.9 (0.4 to 4.8)	12.1* (9.0 to 17.1)
WBC	7.6 (5.5 to 9.9)	8.0 (6.3 to 10.2)	6,5 (5.3 to 9.0)	6.3 (5.2 to 7.1)	8.0 (6.9 to 10.0)	8.2 (6.9 to 11.8)	7.4 (4.9 to 13.6)	6.4 (5.4 to 9.8)	7.7 (5.5 to 8.5)	9.2* (7.9 to 11.2)
LBP	13 (7 to 18)	24* (16 to 30)	8 (5 to 16)	22 (12 to 30)	12 (8 to 17)	16 (10 to 21)	13 (7 to 18)	26* (23 to 29)	15 (10 to 20)	26* (22 to 37)
sTREM-1 serum	149 (108 to 199)	145 (85 to 198)	94 (70 to 282)	90 (76 to 188)	121 (88 to 215)	146 (75 to 180)	145 (97 to 186)	137 (99 to 187)	157 (134 to 188)	204 (109 to 257)

Median (interquartile range) of cytokine levels and soluble triggering receptor on myeloid cells-1 (sTREM-1) values in serum 16 hours and 40 hours following trauma. CRP, C-reactive protein; LBP, lipopolysaccharide binding protein; PCT, procalcitonin; WBC, leucocytes. *P < 0.05 between 16 hours and 40 hours following trauma.

## Discussion

To date, sTREM-1 has been shown to be expressed during pathogen-associated bacterial or fungal pneumonia [7,8]. The role of sTREM-1 during non-infectious inflammation or trauma, however, is not well elucidated. We report here that sTREM-1 is expressed in the alveolar space during the course of non-infectious inflammation due to traumatic lung contusion. We could observe that the expression of sTREM-1 is increased until 40 hours following pulmonary contusion. The severity of lung contusion correlated well with the levels of sTREM-1 in the BAL and the functional impairment of pulmonary function following trauma.

The role of TREM-1 was initially described on neutrophils and monocytes [1]. The authors observe that TREM is activated through lipopolysaccharides present on the surface of bacteria, and that this activation enhances an inflammatory response in an ERK1/2-depenedent and phospholipase-C-dependent fashion. The role of sTREM-1 was then further elucidated during septic shock, identifying its activating role for cytokine release. This activation was associated with an increased serum concentration of sTREM-1 in response to bacterial sepsis. This soluble form was postulated to be increased due to transcriptional activation but the increase could also be due to cleavage from the cellular surface [12]. Furthermore, TREM-1 is known to modulate the innate response either by amplifying or dampening TLR-induced signalling [13]. The *in vitro *inhibition of TREM-1 results in reduced gene expression of the TLR4 pathway, such as the expression of CD14, myeloid differentiation protein-88, IL-10, IL-1β and monocyte chemotactic protein-1 [14]. This inhibition was also implied during bacterial infection in a murine model of pneumococcal pneumonia. The authors postulated that sTREM-1 could hold a protective function for the healing process of the lung [15]. TLR activation, however, is not only initiated by pathogen-associated molecular patterns but also by damage-associated molecular patterns that are released during lung contusion, such as during deceleration or blunt trauma of the lung.

To our knowledge no published data are currently available about the levels of sTREM-1 during non-infectious inflammation of the lung. Evidence about the induction of sTREM-1 in response to non-infectious pathologies was described in the blood of patients suffering from acute pancreatitis without signs of a bacterial infection [16,17]. Recent work has also described that the activation of TLRs can be achieved independently of lipopolysaccharides [18]. The activation of TLRs independent of lipopolysaccharides can result in activation of inflammatory signalling through NF-κB or hypoxia inducible factor [19]. As an adaptive response to tissue trauma or resulting tissue hypoxia, NF-κB-dependent or hypoxia inducible factor-1a-dependent pathways might also be activated [20]. This recently described concept could also be an explanation for non-infectious induction of TREM-1 within tissues. In line with this hypothesis, we report here an induction of sTREM-1 following lung contusion - a non-infectious entity that is associated with tissue hypoxia or TLR activation within the affected pulmonary tissue [20].

The interpretations of the results of the present study are limited by several aspects. First, the sampling of BAL fluid via blind suctioning might enhance the variability of the values measured. This method is established and used in several studies for the diagnosis of ventilator-associated pneumonia [8,21-23] and for the detection of cytokines in the BAL in the context of ventilator-associated lung injury [24]. No data exist, however, on whether the technique of blind suctioning and the collection of samples via bronchoscopy are on a par as far as the measurements of cytokines are concerned.

Furthermore, mechanical ventilation itself stimulates inflammation [25] and might therefore induce increased levels of sTREM-1. Increased sTREM-1 levels could also be explained by bacterial contamination or infection (that is, aspiration on scene). We therefore excluded the BALs of patients positive for intracellular organisms, bacteria or funghi. This approach might be too restrictive, however. since the detection of pathogens in the BAL does not necessarily verify pneumonia. Elevated sTREM-1 levels in the BAL could therefore be theoretically caused by the traumatic injury itself, by inflammation due to mechanical ventilation and by ventilator-associated pneumonia.

Since sTREM-1 levels in the BAL correlate to the severity of trauma, the primary cause of elevated sTREM-1 levels cannot trivially be explained by the mechanical ventilation itself, which in this study followed the same protocol for each patient irrespective of the severity of lung contusion. Furthermore, pneumonia or ventilator-associated pneumonia is not expected to already be present on the day of trauma. This allows us to exclude this reason for sTREM-1 elevation through ventilator-associated pneumonia, since by definition 48 hours of mechanical ventilation are necessary to meet the criteria for ventilator-associated pneumonia.

sTREM-1 also seems to be increased in patients bearing non-infectious processes such as peptic ulcer, inflammatory bowel disease, viral infections, malignant pleural effusions and chronic obstructive pulmonary disease, but also among patients after cardiac surgery or cardiac arrest [4]. Most of the patients included in the study were younger than 40 years of age without relevant comorbidities. One patient anamnestically suffered from asthma bronchiale without daily medical treatment. Complications due to viral infections were not observed within the first 2 days.

Finally, the classification for severity of lung contusion by means of CT scans performed at admission to hospital, which means relatively early after the trauma, might be imprecise. Clinically the severity of lung contusion might differ from radiological visible trauma of the lung in the first hours after trauma. In our population, however, gas exchange parameters correlated negatively with the radiological classified severity of lung contusion. We therefore interpret the classification of lung contusion as a reasonably good indicator for severity of lung contusion in the population investigated.

In summary, the presented data provided evidence that sTREM-1 is expressed during non-infectious inflammation within the lung following traumatic injury. Since the subsequent healing of the lung is to date not well understood, the data from our study indicate sTREM-1 to be an interesting candidate for future investigations into a better understanding of the immunologic processes that are involved after traumatic lung contusion.

## Conclusions

sTREM-1 is known to amplify response to bacterial inflammation (pathogen-associated molecular patterns). In the present article, we demonstrate that sTREM-1 is expressed in the course of nonbacterial inflammation following traumatic lung injury.

## Key messages

• sTREM-1 in BAL is expressed in the course of nonbacterial inflammation following traumatic lung injury

• sTREM-1 in the BAL correlates with both the severity of pulmonary tissue damage (radiological) and functional impairment of gas exchange (FiO_2_/paO_2 _ratio) after traumatic lung injury

## Abbreviations

BAL: bronchoalveolar lavage; CRP: C-reactive protein; CT: computed tomography; ELISA: enzyme-linked immunosorbent assay; ICU: intensive care unit; IL: interleukin; LBP: lipopolysaccharide binding protein; LOS: length of stay; NF: nuclear factor; paO_2_/FiO_2_: arterial oxygen pressure/inspired oxygen fraction; Rs: Spearman correlation coefficient; sTREM-1: soluble triggering receptor on myeloid cells-1; TLR: Toll-like receptor; TREM-1: triggering receptor expressed on myeloid cells-1.

## Competing interests

The authors declare that they have no competing interests.

## Authors' contributions

All authors participated in the study design. TMB, BP, SS and BS participated in data collection. TMB, BP, HW, EVG, IM, KZ and BS analysed and interpreted the data. TMB and BS drafted the report. All authors critically reviewed the report.
